# Engineered hematopoietic stem cells give rise to therapeutic antibody secreting B cells

**DOI:** 10.21203/rs.3.rs-9269825/v1

**Published:** 2026-04-06

**Authors:** Matthew Porteus, Sofia Luna, William Feist, Ashley Utz, Jumana Afaghani, Masashi Miyauchi, Hana Ghanim, Freja Ekman, Anais Amaya, Sridhar Selvaraj, Norman Russkamp, Ludwig Schmiderer

**Affiliations:** Stanford University; Stanford University; Stanford University; Stanford University; Stanford University; Institute for Stem Cell Biology and Regenerative Medicine, Stanford University School of Medicine; Stanford University School of Medicine; Stanford University; Stanford University; Stanford University; Stanford University; Stanford University

## Abstract

Monoclonal antibodies represent half of the top ten selling drugs. Their proven efficacy, however, generally requires repeated administration for prolonged periods of time. In contrast, cell-based therapies offer a different set of pharmacokinetics and pharmacodynamics than traditional medicines, including the potential to have lifetime durability after a single infusion. Here, we describe a genome-engineered stem cell-based platform for continuous antibody production from a single dose. Using CRISPR/Cas9 homology-directed repair mediated editing, we precisely integrated therapeutic antibody expression cassettes into a safe-harbor locus of hematopoietic stem and progenitor cells (HSPCs). Upon differentiation, these gene-targeted HSPCs generate B cells that secrete monoclonal antibodies. We validated this platform using two clinically approved antibodies, achieving efficient targeted integration of the gene-targeted antibodies (GT-Ab) in human HSPCs that successfully engraft in immunodeficient mice. Direct engineering of human B cells demonstrated robust secretion of therapeutic antibodies. To evaluate in vivo antibody production, we transplanted engineered GT-Ab murine HSPCs into immunocompetent mice, achieving durable serum antibody concentrations within the therapeutic range over several months. Lastly, by fusing the antibody to a destabilization domain, we enabled tunable antibody secretion via small molecule regulation. This modular platform establishes a potentially curative approach for chronic diseases currently reliant on repeated antibody administration, offering durable antibody production from a single treatment.

## Introduction

Therapeutic monoclonal antibodies have revolutionized the treatment of a broad spectrum of diseases, including cancer, autoimmune disorders, and infectious diseases. Beginning with the approval of the first monoclonal antibody by the United States Food and Drug Administration (US FDA) in 1986^1^, the number of clinically-approved therapeutic antibodies has grown at an astounding pace that has only accelerated in recent years^2^. As of 2024, there were over one hundred clinically-approved antibodies in the US that act on approximately one hundred distinct pharmacological targets^3^. This has led to the market size of therapeutic antibodies to reach nearly $250 billion in 2023 with a projected growth to >$400 billion by 2028^4^. While therapeutic antibodies have proven to address many needs that prior drug modalities (i.e. small molecules) were unable to, we are now in a new era of cell-based medicines to treat and cure disease. The advantage of cell-based medicines is they have entirely different pharmacokinetic and pharmacodynamic properties compared to traditional medicines, including the ability to proliferate, respond to signals, and migrate into tissues^5^. They also have the potential for long term durability in patients, even for a lifetime if a stem cell is transplanted. With the advances in genome-editing of human cells we can now create a broader class of cell-based medicines by engineering cells with completely new biologic functions. One application of this approach is to modify cells to secrete therapeutic biologic proteins (i.e. antibodies, enzymes, cytokines, hormones). Monoclonal antibodies are a particularly compelling target due to their conserved framework yet variable specificity that supports easy adaption to a broad range of clinical uses.

Despite their proven success, therapeutic antibodies require repeated administration of high bolus doses every 2-4 weeks to maintain their efficacy^6^. Highlighting the need for a long-term delivery platform, there have been several strategies that aim to deliver monoclonal antibodies continuously. First, antibody gene therapy involves the *in vivo* gene transfer of an antibody sequence, most successfully through recombinant viral vectors^7^. Although antibody gene therapy addresses some of the problems associated with traditional antibody injection, there still remain large obstacles such as preexisting immunity to viral vectors and loss of expression over time, which has hampered its clinical development^8^. Cell-based strategies for delivery of antibodies have primarily focused on editing of autologous B cells for antibody expression^9-13^. B cells are an optimal choice for antibody production since they are the immune system’s endogenous specialized antibody secreting lineage. However, engineered B cells face significant translational hurdles as standardized clinical protocols for B cell transplantation are lacking, and long-term evidence on their persistence in humans remains unproven.

Conversely, hematopoietic stem cell transplant (HSCT) is a well-established clinical procedure that has proven to have lifelong durability^14^. Thus, we have developed a genetically engineered cell-based therapy that would enable continuous antibody production from a single dose of autologous hematopoietic stem and progenitor cells (HSPCs). Using CRISPR/Cas9 homology directed repair (HDR) mediated editing, we engineer HSPCs that will give rise to mature B cells capable of secreting a therapeutic antibody of interest. By modifying the HSPC we take advantage of the stem cell’s established ability to engraft and repopulate the blood system^14^ and build on past success of using CRISPR/Cas9 engineered HSPCs for the treatment of disease^15^. However, we aim to restrict antibody expression to the B cell lineage in order to harness its professional ability for antibody secretion – particularly in terminally differentiated plasma cells, which can produce thousands of antibodies per second^16,17^. This strategy also leaves other hematopoietic lineages, including the hematopoietic stem cell (HSC) itself, unperturbed, minimizing any consequences of antibody expression from other cell types. We employ precision CRISPR/Cas9 genome-editing to specifically target the therapeutic antibody expression cassette to a previously established genomic safe-harbor site, *CCR5*^18^, and use a synthetic B cell promoter for antibody expression. By targeting a safe-harbor locus and leaving the endogenous immunoglobulin loci intact, therapeutic antibodies act as passengers in B cell development. We establish proof of concept for this system with two clinically-approved monoclonal antibodies, an anti-PCSK9 antibody for hypercholesterolemia^19^ and an anti-TNFα antibody for autoimmune disease^20^. In this study, we demonstrate high efficiency targeted integration of GT-Abs in human HSPCs that engraft in immunodeficient mice. We then demonstrate that GT-Ab human B cells and transplanted GT-Ab murine HSCs produce strong expression of both therapeutic antibodies *in vitro* and *in vivo*, respectively. Importantly, *in vivo* antibody concentrations reach levels necessary for therapeutic efficacy in humans. Finally, we showcase the ability to regulate antibody expression via a small molecule to improve the safety of this therapy. Altogether, here we show use of a HSPC engineering platform that enables continuous antibody production from derived B cells, offering a versatile system into which any antibody – currently available or developed in the future – could be easily integrated.

## Results

### Efficient targeting of therapeutic antibody expression cassettes to a safe-harbor locus in human HSPCs

The need for repeated administration of therapeutic antibodies led us to develop a genome-engineering strategy enabling continuous cell-based antibody production. In order to express antibodies from a single transcript and reduce mispairing with endogenously expressed antibodies, we utilize a peptide linker to physically pair the light and heavy chain of the antibody^21^. To confirm that functional antibodies can be produced with the linker, each antibody was produced either in the traditional manner using separate plasmids to deliver the light and heavy chain or with one plasmid to deliver the light and heavy chains paired by the peptide linker (Extended Data Fig. 1a). Production of purified antibodies with or without linker was confirmed at the expected molecular weights via western blot analysis for IgG (Extended Data Fig. 1b) and linker antibodies demonstrated comparable activity to their traditional counterparts in antigen-specific functional assays (LDLR expression for anti-PCSK9; TNFa neutralization for anti-TNFa) (Extended Data Fig. 1c,d).

**Figure 1:**
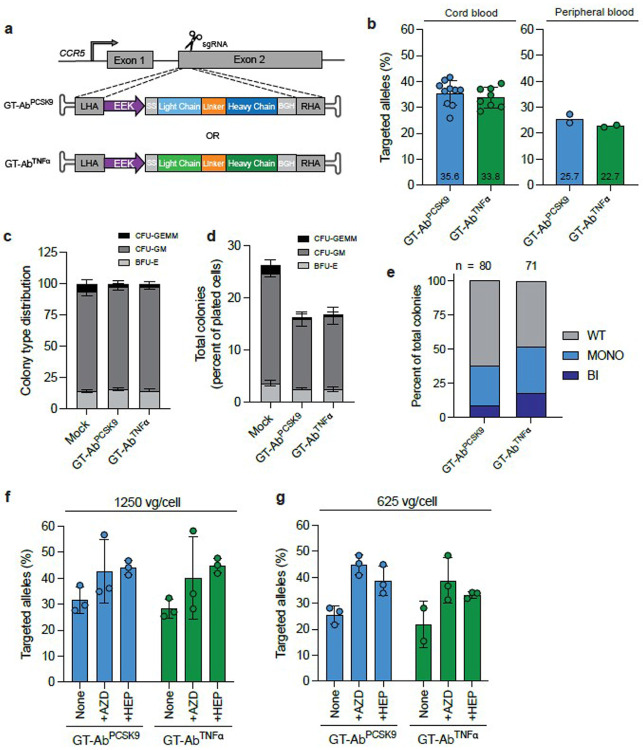
Targeted integration of therapeutic antibody cassettes in CD34^+^ human HSPCs **a.** Schematic of CRISPR/Cas9 genome-editing at *CCR5* locus with delivery of therapeutic antibody cassettes (anti-PCSK9 antibody (GT-Ab^PCSK9^) or anti-TNFα antibody (GT-Ab^TNFα^)) via recombinant AAV6 vector. EEK = EEK promoter, SS = signal sequence, BGH = BGH-polyA signal. **b.** Targeted integration frequency of therapeutic antibody cassettes in cord blood (left) or peripheral blood (right) derived CD34^+^ HSPCs. Dots represent independent biological donors: n = 10 for GT-Ab^PCSK9^ cord blood, n = 8 for GT-Ab^TNFα^ cord blood, n = 2 for both conditions peripheral blood. Mean targeted integration frequency shown at bottom of each bar. **c.** CFU assay of mock edited cells versus GT-Ab edited cells. Bars represent relative frequency of each progenitor colony type: CFU-GEMM (multi-potential granulocyte, erythroid, macrophage, megakaryocyte progenitor cells), CFU-GM (colony forming unit- granulocytes and monocytes), BFU-E (erythroid burst forming units). n=3 independent biological donors, with technical duplicate wells for each donor. **d.** Total colony number for CFU assay described in panel C shown as percent of number of plated cells for each editing condition. n=3 independent biological donors, with technical duplicate wells for each donor. **e.** Frequency of mono- or bi-allelic knock in single cell derived colonies from two of the CFU assays shown in panel C and D. n represents the total number of colonies pooled across two donors. **f.** Targeted integration frequency in CB CD34^+^ cells with no treatment or treatment with 0.5 μM AZD7648 or 5 μM HEP using AAV6 vector dose of 1250 vg/cell. n=3 independent biological donors. **g.** Targeted integration frequency in CB CD34^+^ cells with no treatment or treatment with 0.5 μM AZD7648 or 5 μM HEP using AAV6 vector dose of 625 vg/cell. n=3 independent biological donors for all conditions except n=2 GT-Ab^TNFα^ no treatment. All bars represent mean +/− standard deviation.

We used a single-guide RNA (sgRNA) that specifically targets the human *CCR5* safe harbor locus that was previously validated to achieve high efficiency HDR editing in primary human CD34^+^ HSPCs^22,23^. When used with high-fidelity Cas9^24^ this sgRNA was found to have no measurable insertions/deletions (indels) in the top *in silico* predicted off-target sites^22,23^. To deliver gene-targeted antibody (GT-Ab) donors, adeno-associated virus serotype 6 (AAV6) donor repair templates were designed for therapeutic antibody expression (Fig. 1a). For strong expression in the B cell lineage, antibody expression is driven through a previously described synthetic B cell promoter, the EEK promoter, comprised of human immunoglobulin promoter and enhancer elements^25^. Driving monoclonal antibody expression specifically within the B cell lineage using this promoter has potential efficacy advantages because B lineage cells are the natural producers of high levels of antibody with species-specific glycosylation patterns. It also confers potential safety advantages because the monoclonal antibody will not be produced in other cell types. Primary CD34^+^ cells were edited via electroporation with ribonucleoprotein (RNP) followed by transduction with AAV6 (2500 vector genomes (vg)/cell) carrying the anti-PCSK9 antibody (GT-Ab^PCSK9^) or the anti-TNFα antibody (GT-Ab^TNFα^) sequence. We achieved high targeted integration frequencies of approximately 35% edited alleles in umbilical cord-blood (CB) derived CD34^+^ cells and 24% in adult peripheral blood derived CD34^+^cells in both gene-targeted conditions as measured by in-out droplet digital PCR (ddPCR) (Fig. 1b). To assess the impact of GT-Ab engineering on HSPC proliferation and differentiation capacity, we performed a colony forming unit (CFU) assay and demonstrated distribution of colonies between the major subtypes was not impeded by GT-Ab edited cells relative to mock (electroporation only) edited cells (Fig. 1c). We observed a decrease in total colony formation that was consistent with prior reports of RNP/AAV6 HDR-based editing^26-28^ (Fig. 1d). Measurement of mono- and bi-allelic targeted integration frequencies of each antibody in single-cell colonies revealed that 38% of cells for GT-Ab^PCSK9^ and 52% of cells for GT-Ab^TNFα^ had at least one allele with the therapeutic antibody insertion (Fig. 1e).

Because higher doses of AAV6 are associated with increased activation of the p53 pathway^26^ that has potential to impair HSPC viability and engraftment, we decreased the AAV6 vector dose while maintaining high efficiency editing through use of modulators of the DNA repair pathway. There have been several reported strategies to bias the cell’s endogenous repair pathways following double-stranded break. Small molecule inhibition of non-homologous end-joining (NHEJ) via inhibition of DNA-PKcs with AZD7648 has been shown to increase the frequency of HDR-based editing in multiple primary human cell types, including HSPCs^29^. Alternatively, inhibition of 53BP1, a protein that favors NHEJ by inhibiting the early rate-limiting end-resection step of HDR initiation^30^, has also been reported to increase HDR-based editing^26,31,32^. Thus, we tested the use of either small molecule AZD7648 (AZD) or peptide inhibitor of 53BP1 (HDR Enhancer Protein, HEP) with half (1250 vg/cell) or one-fourth (625 vg/cell) the vector dose of AAV6 in CD34^+^ HSPCs. As expected, lowering of AAV6 vector dose alone led to lower targeted integration frequency and addition of either AZD or HEP led to improvement of targeted integration at both vector doses that reached or exceeded frequencies seen in the original high vector dose condition shown in Fig. 1b (2500 vg/cell) (Fig. 1f,g). Additionally, CFU assay analysis demonstrated that addition of AZD or HEP did not influence HSPC colony distribution (Extended Data Fig. 2a,b). Therefore, to preserve cell health and engraftment potential, we chose to move forward in future experiments using the lowest vector dose tested (625 vg/cell) combined with use of either AZD or HEP to maintain high-efficiency editing.

### GT-Ab engineered HSPCs engraft in immunodeficient mice

**Figure 2:**
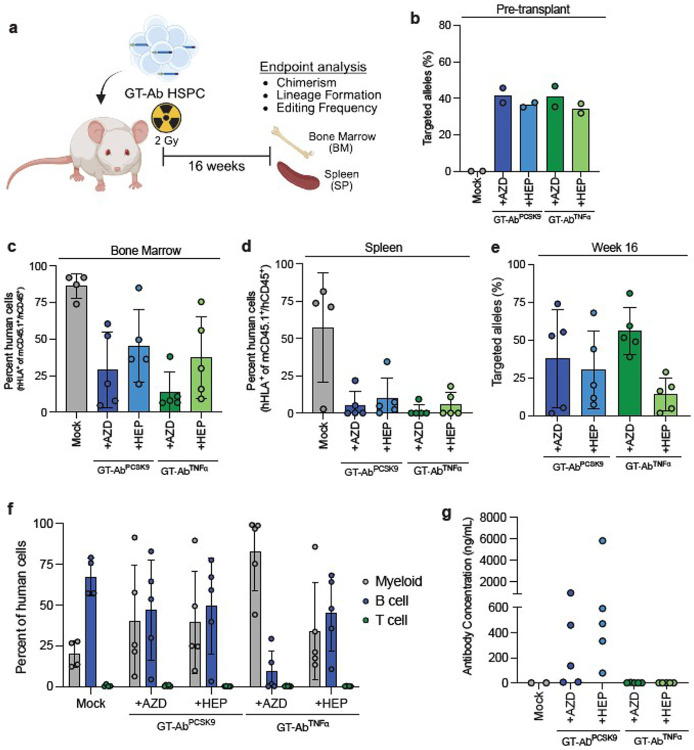
GT-Ab human HSPCs engraft in immunodeficient mice **a.** Graphical representation of transplantation of human HSPCs into NSG mice and endpoint analysis. **b.** GT-Ab targeted integration frequency in CB CD34^+^ HSPCs prior to transplantation. n = 2 donors edited and transplanted separately. **c.** Percent human chimerism in the bone marrow at week 16 posttransplant. Dots represent individual mice: n = 4 mice for Mock, n = 5 mice for each GT-Ab condition. **d.** Percent human chimerism in the spleen at week 16 post-transplant. **e.** GT-Ab targeted integration frequency in bone marrow of mice at week 16 post-transplant. Dots represent individual mice: n = 4 mice for Mock, n = 5 mice for each GT-Ab condition. **f.** Lineage distribution (CD33^+^ myeloid, CD19^+^ B cell, CD3^+^ T cell) in the bone marrow of mice at week 16 post-transplant. Dots represent individual mice: n = 4 mice for Mock, n = 5 mice for each GT-Ab condition. **g.** Serum antibody concentration for each antibody measured by antigen specific ELISA for each antibody at week 16 post-transplant. n = 2 mice for Mock, n = 5 mice for each GT-Ab condition. All bars represent mean +/− standard deviation.

Next, we sought to confirm that our genome engineering strategy does not impair the key function of HSPCs to engraft and reconstitute the blood system. Transplantation of human HSPCs into immunodeficient mice is a powerful tool to evaluate human cell hematopoiesis and is considered the pre-clinical gold standard for evaluating the stem cell potential of cell therapies^33^. We therefore edited two donors of CB CD34^+^ cells with either GT-Ab^PCSK9^ or GT-Ab^TNFα^ using 625 vg/cell of AAV6 and either AZD or HEP. GT-Ab HSPCs were then transplanted into sub-lethally irradiated NSG mice and chimerism and blood cell lineage formation was analyzed after 16 weeks (Fig. 2a). Prior to transplantation, allelic editing frequencies in HSPCs were ~41% in the AZD conditions and ~35% in the HEP conditions (Fig. 2b). At 16 weeks post-transplantation, cells from all GT-Ab conditions successfully engrafted in the bone marrow and showed migration to the spleen, a secondary hematopoietic site (Fig. 2c,d). There was a decrease in engraftment in the GT-Ab conditions compared to mock edited cells, which was consistent with those seen in prior studies using RNP/AAV6 gene-modified cells^28,34,35^ and thus likely not due to the introduction of the antibody cassette itself. Further supporting this point, there was maintenance of GT-Ab^PCSK9^ or GT-Ab^TNFα^ edited cells in the bone marrow of mice at endpoint, indicating gene-targeted HSPCs retain their ability to engraft (Fig. 2e). By using the lower MOI of AAV6, we observed no drop in engraftment of the HDR edited allele frequency from pre-transplant (Fig. 2b) following engraftment (Fig. 2e). There was variability in frequency of targeted integration between mice, but on average the editing frequency (38% in GT-Ab^PCSK9^+AZD, 30% in GT-Ab^PCSK9^+HEP, 56% in GT-Ab^TNFα^+AZD) neared or surpassed that seen in each condition pre-transplant except in one group (14% in GT-Ab^TNFα^+HEP). GT-Ab HSPCs reconstituted the myeloid, B cell, and T cell lineages at similar frequencies to control cells indicating the gene-editing strategy does not introduce major reconstitution bias. There was an observed bias to the myeloid lineage in individual mice that exhibited overall lowered levels of engraftment, consistent with prior reports using this mouse model^22,23,35^ (Fig. 2f). Notably, due to their lack of immune system, immunodeficient mice do not reconstitute with highly differentiated human B cells^36,37^. Likely due to insufficient T cell help, in previous studies we have observed very low or absent levels of human IgG production from xeno-transplanted mice^23^. Thus, when we measured therapeutic antibody concentration in the serum of GT-Ab xeno-transplanted mice via antigen specific ELISA, it was not surprising to see very low levels of anti-PCSK9 antibodies in the GT-Ab^PCSK9^ mice and no anti-TNFα antibodies in the GT-Ab^TNFα^ except a very low amount in one mouse (4.7 ng/mL) (Fig. 2g).

### GT-Ab engineered B cells efficiently secrete therapeutic antibodies

**Figure 3:**
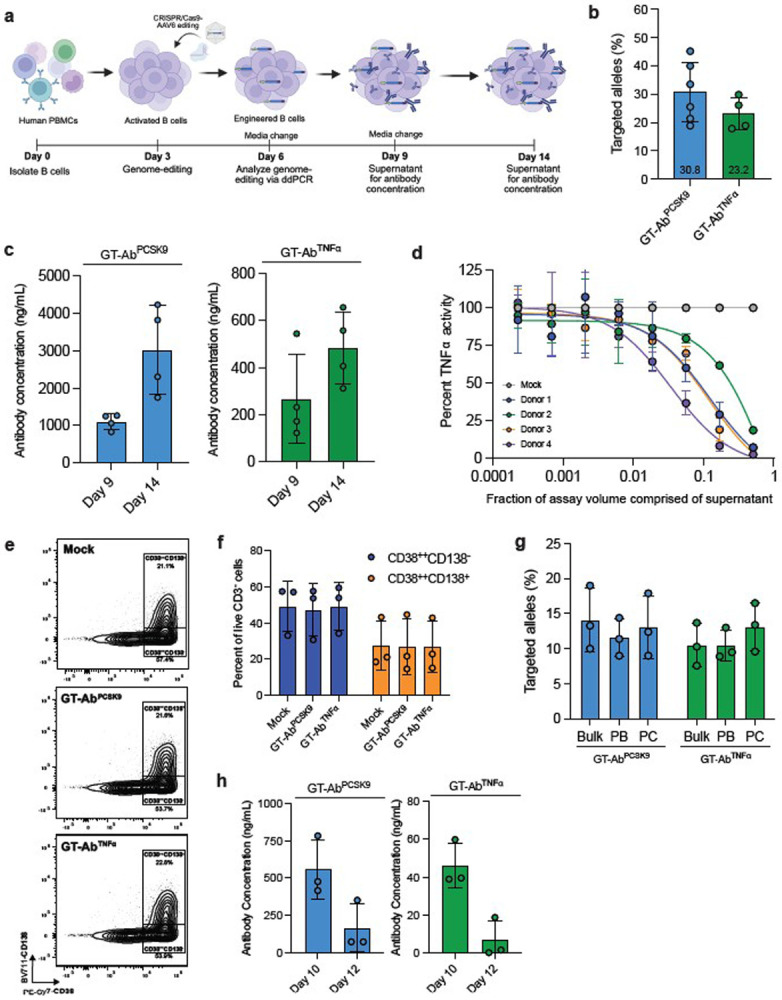
GT-Ab human B cells express functional antibodies and differentiate into plasma cells *in vitro* **a.** Timeline of isolation, genome editing, and media supernatant collection of primary human B cells. **b.** Targeted integration frequency of therapeutic antibody cassettes at *CCR5* locus in primary human CD19^+^ B cells. Cells were targeted with an AAV6 vector dose of 25,000 vg/cell. Dots represent independent biological donors: n = 6 for GT-Ab^PCSK9^ and n = 4 for GT-Ab^TNFα^. Mean targeted integration frequency shown at bottom of each bar. **c.** Antibody concentration in cell culture supernatant at each indicated timepoint as measured by antigen specific ELISA for each antibody. n = 4 for all conditions. **d.** Percent TNFα activity measured by NF-κB reporter assay when supernatant applied from GT-Ab B cells. Each donor activity normalized to mock for same B cell donor. Donor 1 & 2 targeted with an AAV6 vector dose of 10,000 vg/cell and Donor 3 & 4 targeted with an AAV6 vector dose of 25,000 vg/cell. Dots and error bars represent mean and standard deviation of technical duplicates. **e.** Representative flow cytometry plot of mock, GT-Ab^PCSK9^, and GT-Ab^TNFα^ B cells differentiated to plasma cells on day 12. **f.** Frequency of CD38^++^CD138^−^ plasmablasts and CD38^++^CD138^+^ plasma cells of live single CD3^−^ cells for three primary B cell donors. **g.** Targeted integration frequency in bulk cells versus FACS isolated CD38^++^CD138^−^ plasmablasts (PB) and CD38^++^CD138^+^ plasma cells (PC) on day 12. n = 3 for all conditions. **h.** Antibody concentration in cell culture supernatant as measured by antigen specific ELISA for each antibody. n = 3 for all conditions. All bars represent mean +/− standard deviation.

Given the limitations in B cell development of transplanted human HSPCs in immunodeficient mice, direct editing of primary human B cells offers a potential alternative for modeling gene-targeted antibody secretion. CD19^+^ B cells from healthy adult peripheral blood mononuclear cells (PBMCs) were isolated and activated for three days using a previously published protocol^11^. We then used the same GT-Ab engineering system described above to edit the activated B cells and measured targeted integration frequency and antibody concentration in the culture supernatant after varying amounts of time in culture (3 and 5 days) (Fig. 3a). At three days post-editing, we observed targeted integration frequencies in the B cells that was comparable to those seen in CD34^+^ cells (average 30.8% for GT-Ab^PCSK9^ and 23.2% for GT-Ab^TNFα^) (Fig. 3b). At day 9 post-activation after leaving cells for 3 days in culture, we observed efficient secretion of either anti-PCSK9 or anti-TNFα antibodies into the culture supernatant measured by antigen specific ELISA for each antibody (Fig. 3c). After replating and a five-day incubation period, we observed an expected increase in antibody concentration in both conditions (Fig. 3c). Interestingly, anti-PCSK9 antibodies were produced more efficiently than anti-TNFα antibodies, potentially reflecting differences in half-life of antibodies or the use of more contemporary antibody engineering approaches in the development of the anti-PCSK9 antibody. To demonstrate that antibodies produced from gene-targeted B cells were functional, we applied the supernatant from the GT-Ab^TNFα^ B cells to a TNFα activity assay using a THP-1 NF-κB reporter cell line^38^. GT-Ab^TNFα^ B cell supernatant collected from four biological donors was able to efficiently eliminate TNFα activity compared to supernatant from mock edited cells (Fig. 3d).

Next, we investigated if GT-Ab B cells were able to differentiate into plasma cells, the terminally differentiated antibody secreting cell type, capable of secreting thousands of antibodies per cell per second^17,39^. Using a previously defined protocol^12^, GT-Ab B cells were successfully differentiated into CD38^++^CD138^-^ plasmablasts and CD38^++^CD138^+^ plasma cells at similar frequencies to control cells (Fig. 3e,f). Plasmablasts and plasma cells were then isolated via fluorescence activated cell sorting (FACS) and genomic DNA was isolated from each population. Analysis of editing in each population by ddPCR revealed maintenance of GT-Ab edited alleles in both populations compared to the bulk population, indicating that there is no selection against edited cells (Fig. 3g). Notably, differentiated cells were able to secrete antibodies with measured concentrations highest at day 10 of differentiation (Fig. 3h). Altogether, these results support the idea that B cells carrying the GT-Ab edit can successfully secrete therapeutic antibodies and differentiate into plasma cells.

### Transplanted GT-Ab mouse HSPCs achieve therapeutic antibody concentrations

**Figure 4:**
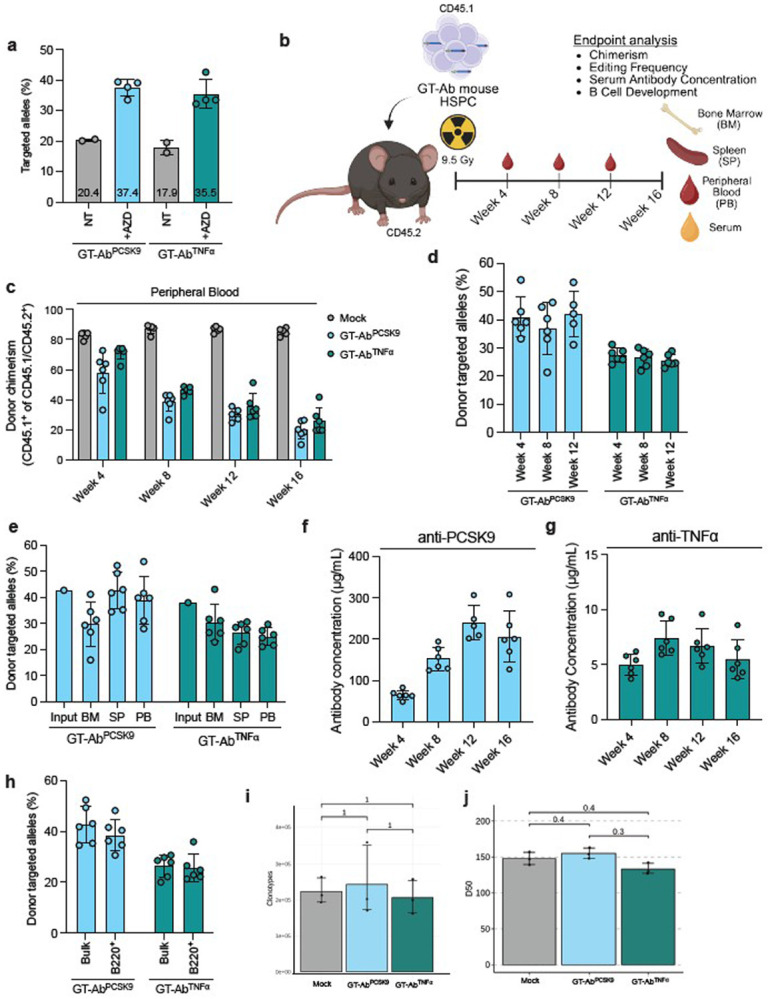
GT-Ab engrafted immunocompetent mice produce therapeutically relevant concentrations of antibodies **a.** Targeted integration frequency of therapeutic antibody cassettes at *Rosa26* locus in murine HSPCs with and without 1 μM AZD7648. NT = no treatment. Dots represent independent murine HSPC cultures: n = 2 for GT-Ab with no treatment, n = 4 for GT-Ab +AZD. Mean targeted integration frequency shown at bottom of each bar. **b.** Graphical representation of timeline of transplantation, blood collection, and endpoint analysis in C57BL/6. **c.** Donor chimerism (CD45.1^+^ of total CD45) in peripheral blood at week 4, 8, 12, and 16. Dots represent individual mice: n = 5 mice for Mock, n = 6 mice for each GT-Ab condition except in week 12 GT-Ab^PCSK9^ n = 5 due to insyffucient blood collection in one mouse. **d.** Targeted integration frequency of GT-Abs in peripheral blood at week 4, 8, and 12. Values calculated by dividing total targeted integration frequency measured by ddPCR by donor chimerism measured by flow cytometry in panel C. n are same as in panel c. **e.** Targeted integration frequency of GT-Abs in bone marrow (BM), spleen (SP), and peripheral blood (PB) at week 16 compared to targeted integration frequency of HSPCs prior to transplantation (input, n=1). Values for BM, SP, and PB calculated as in panel D. Dots represent individual mice: n = 5 mice for Mock, n = 6 mice for each GT-Ab condition. **f.** Serum antibody concentration of anti-PCSK9 antibody measured by antigen specific ELISA in GT-Ab^PCSK9^ transplanted mice at week 4, 8, 12, and 16. n are same as in panel C. **g.** Serum antibody concentration of anti-TNFα antibody measured by antigen specific ELISA in GT-Ab^TNFα^ transplanted mice at week 4, 8, 12, and 16. n are same as in panel C. **h.** Targeted integration frequency in bulk spleen cells versus FACS isolated B220^+^ B cells at week 16 post-transplant. n= 6 mice per condition. Data for bulk same as in panel E. i. Total number of unique clonotypes identified by analysis of mouse BCR sequencing. n = 3 mice per condition. Bars represent mean +/− 2.5-9.75% quantile range. P = 1 for comparison between each group by Kruskal–Wallis test adjusted for multiple testing using the Holm–Bonferroni correction. **j.** D50 diversity score from analysis of clonotypes from mouse BCR sequencing. n = 3 mice per condition. Bars represent mean +/− 2.5-9.75% quantile range. P = 0.4 for Mock v. GT-Ab^PCSK9^ and Mock v. GT-Ab^TNFα^, P = 0.3 for GT-Ab^PCSK9^ v. GT-Ab^TNFα^ by Kruskal–Wallis test adjusted for multiple testing using the Holm– Bonferroni correction. All bars represent mean +/− standard deviation unless noted otherwise.

To better evaluate *in vivo* antibody production, we modeled autologous HSCT through transplantation of murine GT-Ab HSPCs into immunocompetent mice, capable of reconstituting with fully mature antibody secreting B cells. To accomplish this, murine c-kit^+^ HSPCs were isolated from bone marrow of CD45.1 C57BL/6 mice and expanded ex vivo, using a protocol adapted from previously published protocols^40,41^. Following expansion, we employed a sgRNA targeting the murine *Rosa26* safe harbor locus^42-44^ and redesigned the AAV6 repair donors containing the antibody cassettes for integration at this locus (Extended Data Fig. 3a). To reach editing frequencies in murine HSPCs similar to that achieved in human HSPCs we tested use of AZD (Extended Data Fig. 3b) and found that 1 μM AZD was able to increase targeted integration frequencies to those achieved in human HSPCs (Fig. 4a).

Using this optimized editing protocol, 1x10^6^ CD45.1 GT-Ab murine HSPCs along with 2.5x10^5^ CD45.1/CD45.2 bone marrow support cells were transplanted into lethally irradiated CD45.2 C57BL/6 mice and peripheral blood was taken every four weeks until endpoint at 16 weeks post-transplant (Fig. 4b). Prior to transplant, cells were assessed for frequency of c-Kit^+^Sca1^+^Lineage^-^ (KSL) HSPCs and we found a slight decrease in frequency of KSL cells in the GT-Ab conditions compared to mock (Extended Data Fig. 3c,d). We observed engraftment of mock and GT-Ab HSPCs in the peripheral blood at week four post-transplant (average 82.8% for mock, 57.9% for GT-Ab^PCSK9^, and 71.5% for GT- Ab^TNFα^), which decreased at week 8 and then remained relatively stable through week 16 for the GT-Ab conditions (Fig. 4c). Consistent that gene editing causes some toxicity to HSPCs^44^, endpoint analysis of bone marrow showed lower engraftment of GT-Ab HSPCs (average chimerism 8.3% for both GT-Ab^PCSK9^ and GT-Ab^TNFα^) compared to mock edited cells (average 79.9%). This pattern was also consistent although less prominent in the spleen (average chimerism 83.3% for Mock versus 19.5% for GT-Ab^PCSK9^ and 29.4% for GT-Ab^TNFα^) (Extended Data Fig. 4a). We also observed that donor lineage reconstitution (B cell, T cell, myeloid) in the spleen and peripheral blood was similar when comparing mock and GT-Ab conditions. In the bone marrow, likely due to the very low levels of donor engraftment, we saw bias toward the B cell lineage in the GT-Ab conditions, although all three hematopoietic lineages were still able to form (Extended Data Fig. 4b). Importantly, we saw stable maintenance of GT-Ab edited alleles compared to input editing in the peripheral blood at each timepoint (Fig. 4d) and in all three hematopoietic compartments at endpoint when accounting for donor chimerism (Fig. 4e), indicating that there is no selection against the GT-Ab edited cells following engraftment.

To evaluate antibody secretion from transplanted cells, serum samples were collected every four weeks and run on antigen specific ELISA. We measured strong expression of each antibody as early as four weeks post-transplant with concentrations increasing until 12 weeks and maintaining to endpoint at week 16 (Fig. 4f,g). Consistent with *in vitro* data in GT-Ab human B cells, we saw the anti-PCSK9 antibody was produced much more efficiently than the TNFα antibody in the serum of mice. At week 16, the anti-PCSK9 antibody reached an average concentration of 206 μg/mL while the anti-TNFα antibody reached an average concentration of 5.5 μg/mL. Nevertheless, both antibodies either reached or far surpassed concentrations necessary for therapeutic efficacy in humans for each respective antibody (approximate therapeutic concentration 10-15 μg/mL for anti-PCSK9 and 3-7 μg/mL for anti-TNFα antibody) ^45,46^. We also assessed mouse IgG production in the transplanted mice and found there was an average of ~1600 μg/mL of mouse IgG at 16 weeks. Thus anti-PCSK9 and anti-TNFα antibodies represented an average 13.1% and 0.4% of total IgG, respectively (Extended Data Fig. 4c). Of note, due to the lower chimerism observed in the GT-Ab mice, these serum antibody concentrations were achieved with only 3.2-18.5% total edited cells in the bone marrow when accounting for editing frequency and chimerism, which resulted in 4.3-13.8% edited cells in the spleen and 3.1-13.9% edited cells in the peripheral blood. The fact that robust antibody production can be achieved despite low levels of edited cell chimerism indicates that our approach may be effective even with low levels of engraftment. This suggests that durable therapeutic antibody production could be established without the need for aggressive myeloablative conditioning, thereby improving the safety and clinical feasibility of this strategy.

We next characterized B cell development in mice transplanted with GT-Ab murine HSPCs to evaluate for any perturbations. First, we performed flow cytometry to phenotypically characterize B cells in the bone marrow and spleen. In the bone marrow, we observed similar frequencies of B220^low^ pre-pro-B, pro-B, and pre-B cells in the bulk population, but observed an increase in pre-pro-B cells in the GT-Ab conditions in the donor CD45.1^+^ cells alone which may reflect artifact of low donor engraftment frequencies (Extended Data Fig. 5a). In the spleen, we looked for IgD^+^ mature B cells and found that mock and GT-Ab cells formed similar frequencies of both early mature (IgD^+^IgM^+^) and late mature (IgD^+^IgM^-^) B cells in both the bulk population and donor CD45.1^+^ cells alone (Extended Data Fig. 5b). We then isolated the B220^+^ donor-derived spleen cells by FACS and found that GT-Ab targeted integration frequencies were comparable to that of the bulk population, indicating no selection against the edited cells during formation of B cells (Fig. 4h). To more carefully interrogate the development of GT-Ab B cells in vivo, we performed B cell receptor (BCR) sequencing on the donor B220^+^ spleen cells to evaluate for any perturbation in the BCR repertoire. We found a similar number of clonotypes and length of CDR3 regions between groups (Fig. 4i, Extended Data Fig. 5c). We also found that the number of clones occupying the top 50% of total sequencing reads (D50 diversity index) was similar amongst all groups, indicating similar BCR diversity (Fig. 4j). Similarly, there was no significant differences in V/J gene usage (Extended Data Fig. 5d,e). Taken together, these data largely support that introduction of the GT-Ab cassette in a safe-harbor locus and expression of exogenous antibodies does not impair B cell formation or development in vivo.

### Engineered antibody levels can be modulated through small molecule regulation

**Figure 5:**
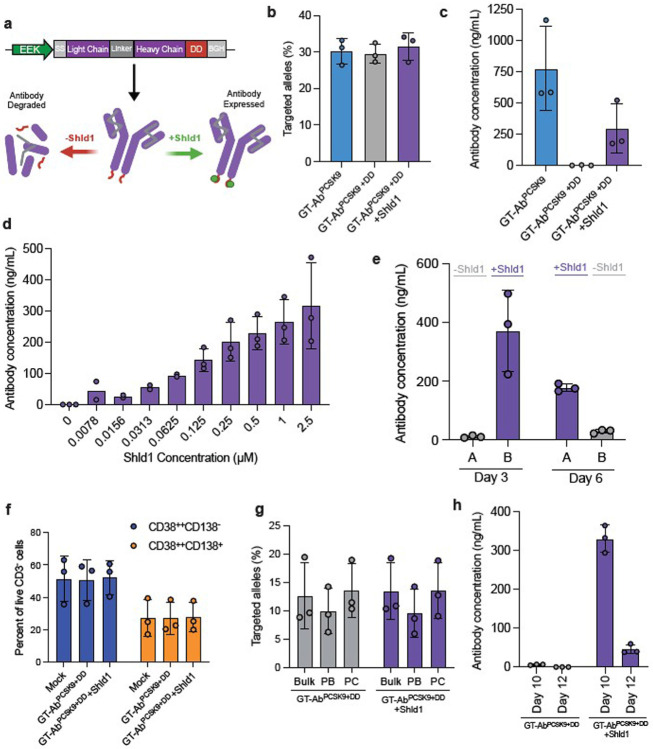
Engineered antibody expression can be tuned and reversed via small molecule regulation **a.** Schematic of gene-targeted antibody fused to FKBP12 destabilization domain (DD) at the C-terminus and resultant proteins products with and without ligand, Shield-1 (Shld1). **b**. Targeted integration frequency of anti-PCSK9 antibody or anti-PCSK9 antibody +DD cassette at *CCR5* locus in primary human CD19^+^ B cells. Dots represent independent biological donors: n = 3 for each condition. **c.** Antibody concentration in cell culture supernatant three days post-editing as measured by antigen specific ELISA for the anti-PCSK9 antibody. n = 4 for all conditions. **d.** Antibody concentration in cell culture supernatant three days post-editing as measured by antigen specific ELISA for each antibody. n = 2 for 0.0078-0.0625 μM, n = 3 for all other concentrations. **e.** Antibody concentration with (purple) and without (gray) Shld1 three days post-editing. Cells were then switched groups (−/+Shld1) and incubated for an additional three days, n = 3. **f.** Frequency of CD38^++^CD138^−^ plasmablasts and CD38^++^CD138^+^ plasma cells of live single CD3^−^ cells for three primary B cell donors. **g.** Targeted integration frequency in bulk cells versus FACS isolated CD38^++^CD138^−^ plasmablasts (PB) and CD38^++^CD138^+^ plasma cells (PC) on day 12, n = 3. h. Antibody concentration in cell culture supernatant with and without addition of Shld-1 as measured by antigen specific ELISA, n = 3. All bars represent mean +/− standard deviation.

The high levels of antibodies seen in the immunocompetent mouse model prompted us to develop a system in which antibody expression could be regulated. Previous work by the Wandless group developed a system to tune protein expression through fusion of a protein of interest to a FK506 binding protein 12 (FKBP12) destabilization domain (DD)^47^. The protein fused to the DD is rapidly degraded unless a synthetic ligand, Shield-1 (Shld1), is provided to stabilize the DD. We designed an AAV6 repair template containing the anti-PCSK9 antibody expression cassette with the DD fused to the C-terminus of the heavy chain of the antibody (Fig. 5a, Extended Data Fig. 6a). We then targeted primary human B cells with the antibody alone (GT-Ab^PCSK9^) or with the antibody fused to the FKBP12-DD (GT-Ab^PCSK9+DD^). Despite similar targeted integration frequencies in each group (Fig. 5b), addition of the FKBP12-DD led to decreased antibody expression even in the presence of Shld1 (Fig. 5c). Nonetheless, to demonstrate that antibody levels can be modulated using this system, we applied varying concentrations of Shld1 to GT-Ab^PCSK9+DD^ edited cells. Measurement of antibody concentration in the supernatant revealed that expression could indeed be finely tuned through Shld1 concentration (Fig. 5d, Extended Data Fig. 6b). Lastly, we assessed whether antibody expression is reversible using the FKBP12-DD. GT-Ab^PCSK9+DD^ B cells were split into a +Shld1 (Group A) and -Shld1 (Group B) condition and after three days, antibody expression was almost exclusively seen in the +Shld1 condition (Group A). To evaluate reversibility, we then switched the groups and checked antibody concentration in the supernatant after three days. We observed little antibody expression in the cells with Shld1 removed (Group A), while the introduction of Shld1 to Group B led to new antibody expression (Fig. 5e). Lastly, we demonstrate that antibody secretion can also be controlled using the FKBP12-DD in human B cells differentiated to plasma cells (Fig. 5f-h). Altogether, these results demonstrate that addition of the FKBP12-DD allows for finely tunable and reversible antibody expression. The ability to precisely regulate antibody levels may be critical for the safe delivery of sustained antibody production using cell-based therapies.

## Discussion

Although hematopoietic stem cell transplantation has been a curative therapy for decades, advances in genome-engineering allow us to expand the scope for which it may be useful. The FDA approval of the first CRISPR edited HSC medicines in 2024^48^ marked a major step forward in using genome-engineering to treat disease. While autologous HSCT has traditionally focused on curing genetic diseases, our work expands this paradigm, demonstrating that genome-edited HSCs could effectively treat non-genetic conditions by re-writing new function into cells. Here we explore one application of this ability by using HDR-based genome-editing of HSPCs to introduce therapeutic antibody expression in the B cell lineage. We first show efficient targeted integration frequency of up to 40% GT-Ab targeted alleles in human CD34^+^HSPCs. Because RNP/AAV6-based editing has been shown to impact the health and engraftment ability of HSPCs^49-51^, we next lowered the amount of AAV6 used in conjunction with modulators of the DNA repair pathway to maintain high-efficiency editing. Using either inhibition of NHEJ (AZD7648) or inhibition of 53BP1 (HEP), we considerably lowered the vector dose of AAV6 while maintaining ~40% GT-Ab targeted alleles (Fig. 1). We next demonstrated that GT-Ab human HSPCs successfully engraft in immunodeficient mice, with edited alleles maintained at an average of ~35% across conditions in the bone marrow (Fig. 2). Although we saw minimal antibody expression in the xenografted mice due to their limited ability to form antibody secreting B cells, we then turned to two alternate models to evaluate antibody expression. First, we show that GT-Ab B cells produce functional antibodies, and these B cells can mature into plasma cells *in vitro* (Fig. 3). Next, we model autologous HSCT in an immunocompetent setting by engineering and transplanting GT-Ab murine HSPCs which led to strong production of therapeutically relevant concentrations of antibodies *in vivo* with no apparent disruption in normal B cell development (Fig. 4). Lastly, by fusing the gene-targeted antibody to a destabilization domain, we demonstrate that antibody concentration can be controlled through small molecule modulation (Fig. 5).

The use of cells as drug delivery vehicles has long gained attention due to the potential to harness the intrinsic pharmacokinetic and biodistribution properties of different cell types^52^. Yet somatic cell-based therapies are unlikely to rival the lifelong durability of HSCT. Additionally, past efforts have relied on transient modification of cells, such as through drug encapsulation or attachment on the cell surface^53^. With advances enabling the stable integration of large genetic payloads into a variety of primary cell types^54^, it is now possible to achieve sustained, long-term expression of therapeutic proteins. A permanently genetically engineered stem cell confers ideal properties for the stable production of drugs through a single dose, unlike any other medicine developed before.

In this work, we utilize RNP/AAV6 HDR-mediated editing as AAV6 remains one of the most efficient methods of donor DNA delivery to HSPCs. Despite this, it is well known that the use of AAV6 impacts the ability HSPCs to engraft^22,23,28^, which is primarily due to the activation of the DNA damage response and p53 pathway^26,55^. Yet, much work is currently underway to ameliorate these effects. Here we explore some of these methods by reducing the vector dose of AAV6 in combination with NHEJ or 53BP1 inhibitors, which has been previously shown to reduce toxicity in HSPCs^26,29,32^. Alternatively, a range of DNA donor delivery approaches—including single-stranded oligodeoxynucleotides (ssODNs), lipid nanoparticles, and integrase-deficient lentiviral vectors (IDLVs)^50,56^—are under active investigation for reducing toxicity. As these strategies continue to mature, the antibody engineering platform described here could be readily incorporated into alternative delivery vectors.

Past work has investigated the direct use of human B cells and plasma cells to deliver therapeutic proteins, including antibodies^9-13,57^. While we believe the transplantation of engineered HSPCs has the potential for longer lasting durability than this approach, we recognize the choice of B cells specifically to express the therapeutic antibody to be important. We therefore forego the use of a ubiquitous promoter that might express in many off-target lineages in favor of the EEK promoter that limits expression to the B cell lineage to limit any unintended effects on other cell types. Additionally, as the endogenous antibody-secreting lineage, B cells offer several advantages over other cell types. First, terminally differentiated plasma cells are capable of secreting antibodies at an enormous rate (10^2^-10^3^ antibodies per second per cell)^16,17^. In our approach, the frequency of engineered HSPCs in the bone marrow should mirror the frequency of engineered B cells in the periphery. Use of a pan-B cell promoter drives antibody expression across all B cell sub-types, including plasma cells, allowing for robust antibody secretion proportional to overall B cell reconstitution. In addition to producing high levels of antibodies, B cells may endow beneficial natural post-translational modifications to their antibody products, particularly since IgG antibodies undergo extensive N-glycosylation following translation^58^. Glycosylation of antibodies is known to effect immunogenicity, function, and longevity^59,60^, thus B cell produced antibodies have the potential to be longer lived and less immunogenic than their traditionally injected counterparts. Because expression from B cells is likely to be most advantageous, future work may benefit from exploring methods of more specific B cell lineage expression than use of a synthetic promoter, such as knock-in downstream of a B cell specific gene, co-opting an endogenous promoter.

Despite the grueling repeated injection or infusion regimens, it is unlikely that patients with diseases that currently require injections of therapeutic antibodies would be subjected to the toxic chemotherapeutic pre-conditioning that is currently the standard of care prior to HSCT. Rather, we envision this therapy to be paired with reduced-intensity, non-toxic conditioning regimens. Currently, strategies to employ antibody-based conditioning which selectively target and deplete HSCs are in both pre-clinical work and clinical trials^61-64^. Notably, in our model of autologous HSCT in immunocompetent mice the GT-Ab mice had an average of only 2.4% edited alleles in the bone marrow at endpoint which resulted in therapeutically relevant antibody concentrations in the bloodstream (on the scale of micrograms/milliliter), highlighting the potential compatibility of this therapy with reduced-intensity pre-transplant conditioning.

Although two clinically relevant monoclonal antibodies were used in this work as a proof-of-concept, it is important to note that this platform’s modular nature allows for easy swapping of monoclonal antibodies for current and future targets. Furthermore, as antibody-like proteins (such as nanobodies^65^, heavy-chain antibodies (HCAbs)^66^, and bispecific T cell engagers (BITEs)^67^) continue to be developed, these therapeutics could also readily be implemented in our system. Further, while antibodies are a natural first drug to be produced by B cell progeny, more broadly, this system has the potential to deliver any protein biologic. Many biologics, especially those of small size, currently suffer from short half-lives due to rapid kidney filtration or cleavage by peptidases necessitating frequent, taxing dosing regimens for patients^68^. Fusion proteins (e.g. etanercept), enzymes (e.g. factor IX, lysosomal enzymes), and peptides (e.g. semaglutide) could all potentially benefit from inclusion in this system. Overall, the work presented here provides a framework for sustained delivery of therapeutic protein biologics from a single infusion of HSPCs.

## Methods

### Antibody production and purification.

Full length heavy and light chains for each antibody were cloned by restriction enzyme digest or Gibson Assembly (New England Biolabs) into pCMVR either individually or with a linker. Antibody constructs were expressed in Expi293F cells (Thermo Fisher Scientific, cat.: A14527) and grown in combined media (66% FreeStyle/33% Expi media, Thermo Fisher Scientific, cat.: 12338018 and A1435101, respectively) at 37 °C and 8% CO2 with shaking at 120 rpm. The day of transfection, cells were diluted to a density of approximately 3-4 × 10^6^ cells per ml. For a 100 mL transfection volume: 60 μg of total DNA was added to 10 mL of expression media, followed by dropwise addition of 130 μL of FectoPRO transfection reagent (Polyplus, Sébastien Brant, France, cat.: 101000014) with vigorous mixing. Heavy and light chain plasmids were used at a 1:1 ratio, while single plasmid linker antibody constructs were used alone. For simultaneous production of a traditional and linker antibody, 20 μg of each plasmid (traditional light chain, traditional heavy chain, and linker antibody) were applied in 100 mL cell cultures. After a 10-minute incubation at room temperature, the transfection mixture was added to 100 ml of cells. D-glucose (4 g/L, Sigma-Aldrich) and valproic acid (3 mM, Acros Organics) were then added to the cells immediately post-transfection to increase recombinant protein production. The cells were harvested 3–5 days after transfection by spinning the cultures at 7,000g for 5 minutes. Cell culture supernatants were filtered through a 0.22μm filter, combined with 1/10th volume of 10x HEPES-buffered saline (HBS) and purified using the ÄKTA pure fast performance liquid chromatography (FPLC, Cytiva) with a 5 mL MabSelect PrismA column (Cytvia, cat.: 17549802). The ÄKTA system was equilibrated with: A1 – 1x HBS; A2 – 100 mM glycine pH 2.8; B1 – 0.5 M NaOH; Buffer line – 1x HBS; and Sample lines – ddH2O. The column was washed with A1 and then the proteins were eluted with A2 into 50mL conical tubes containing 2.5 mL of 1M HEPES buffer, pH 7.4. The column was then washed with A1, B1 and A1. The resultant Fc-containing samples were buffer-exchanged and concentrated using 50-kDa or 100-kDa cutoff centrifugal concentrators. The samples were further purified by size exclusion on the same ÄKTA FPLC with Superdex 200 Increase 10/ 300 GL column (Cytiva, cat.: 28-9909-44) and further concentrated using 50-kDa or 100-kDa cutoff centrifugal concentrators. 10% glycerol was added and protein concentration was determined by absorbance at 280 nm (A280) on a NanoDrop UVVis spectrophotometer. The purity was assessed by protein gel electrophoresis.

### rAAV6 vector design, production, and purification.

Recombinant donor vectors were cloned from a gBlock Gene Fragments (Integrated DNA Technologies, IDT, San Jose, CA, USA) using restriction enzyme digest or Gibson Assembly (New England Biolabs, Ipswich, MA, USA) into an Adeno-associated virus, serotype 6 (AAV6) vector plasmid derived from the pAAV-MCS plasmid (Agilent Technologies, Santa Clara, CA, USA). rAAV6 vectors were either produced in-house or purchased from SignaGen Laboratories (Frederick, MD, USA), PackGene Biotech (Houston, TX, USA) or VectorBuilder Inc. (Chicago, IL, USA). For in-house production, 10-12×10^6^ HEK-293T cells per plate were seeded in ten 15 cm^2^ dishes. After 24-48 hours, each dish was transfected with a standard polyethylenimine (PEI) transfection using 6 μg ITR-containing plasmid and 22 μg pDGM6 plasmid (gift from David Russell, University of Washington, Seattle, WA, USA). After 48-72 hour incubation, cells were harvested and vectors were purified using the AAVpro purification kit (cat.: 6666; Takara Bio, Kusatsu, Shiga, Japan) as per manufacturer’s instructions. Viral titers were determined using droplet digital PCR (ddPCR) to measure the number of vector genomes per microliter as previously described^69^.

### CD34^+^ HSPC isolation and culture.

Human cord-blood CD34^+^ HSPCs were isolated from cord blood by the Stanford Binns Program for Cord Blood Research. Samples were obtained with approval from the Stanford Institutional Review Board Committee under protocol 33813. Briefly, isolated mononuclear cells were positively selected for CD34 using the CD34_+_ MicroBead Kit Ultrapure (Miltenyi Biotec, San Diego, CA, cat.: 130-100-453). Peripheral blood CD34^+^ HSPCs were purchased from AllCells (Alameda, CA) or the Fred Hutchinson Cancer Center (Seattle, WA). Cells were cultures at 1.5×10^5^–2.5×10^5^ cells/mL in CellGenix^®^ GMP Stem Cell Growth Medium (SCGM, CellGenix, Freiburg, Germany, cat.: 20802-0500) supplemented with a human cytokine (PeproTech, Rocky Hill, NJ, USA) cocktail: stem cell factor (100 ng/mL), thrombopoietin (100 ng/mL), Fms-like tyrosine kinase 3 ligand (100 ng/mL), interleukin 6 (100 ng/mL), streptomycin (20 mg/mL), and penicillin (20 U/mL), and 35 nM of UM171 (APExBIO, Houston, TX, USA, cat.: A89505), as previously described^70^. Cells were cultured at 37°C in a hypoxic incubator with 5% CO_2_ and 5% O_2_.

### B cell isolation and culture.

B cells were isolated by negative selection using the human B Cell Isolation Kit II (Miltenyi Biotec, cat: 130-091-151) from leukoreduction system (LRS) chambers obtained deidentified from the Stanford Blood Center. Cells were cultured in Iscove’s Modified Dulbecco’s Medium (IMDM) (Thermo Fisher Scientific, Waltham, MA, USA, cat.: 12440053) supplemented with 10% bovine growth serum (Cytiva, Marlborough, MA, USA, cat.: SH30541.03HI), 1% penicillin-streptomycin (Cytiva, cat.: SV30010), 55 μM of 2-mercaptoethanol (Sigma-Aldrich, St. Louis, MO, USA, cat.: M3148), 50 ng/mL of IL-2 (Peprotech, cat.: 200-02), 50 ng/mL of IL-10 (Peprotech, cat.: 200-10), 10 ng/mL of IL-15 (Peprotech, cat.: 200-15), 100 ng/mL of recombinant human MEGACD40L (Enzo Life Sciences, Farmingdale, NY, USA, cat.: ALX-522-110-C010), and 1 μg/mL of CpG oligonucleotide 2006 (Invivogen, San Diego, CA, USA, cat.: tlrl2006-1) at a density of 1×10^6^ cells/mL at 37 °C, 5% CO_2_, and ambient oxygen levels, as described previously^11^.

### Plasma cell differentiation and characterization.

B cells were isolated as described above and then activated and differentiated as described previously^71^. Briefly, cells were cultured in a base media of Iscove’s Modified Dulbecco’s Medium (IMDM) (Thermo Fisher Scientific, cat.: 12440053) supplemented with 10% bovine growth serum (Cytiva, cat.: SH30541.03HI), 55 μM of 2-mercaptoethanol (Sigma-Aldrich, cat.: M3148). For the first seven days, media was supplemented with 100 ng/mL of recombinant human MEGACD40L (Enzo Life Sciences, cat.: ALX-522-110-C010), 1 μg/mL of CpG oligonucleotide 2006 (Invivogen), and 40 ng/mL IL-21 (Peprotech, cat.: 200-21). For days 7-12, media supplementation was changed to 50 ng/mL IL-6 (Peprotech, cat.: 200-06), 10 ng/mL of IL-15 (Peprotech, cat.: 200-15), and 15 ng/mL interferon-α2B (Sigma-Aldrich, cat.: H6166). Cells were plated at a density of 1-1.5 × 10^6^ cells/mL at 37 °C, 5% CO_2_, and ambient oxygen levels. To evaluate differentiation, cells were blocked for non-specific binding using FcR Blocking Reagent for human (Miltenyi Biotec, cat.: 130-059-901) 10 min at room temperature and then stained for 20 min at 4°C with the following antibody cocktail: anti-human CD38 (BD Biosciences, cat.: 560677), anti-human CD3 (Biolegend, cat.: 300408), anti-human CD19 (Biolegend, cat.: 302211), anti-human CD138 (Biolegend, cat.: 356521), and viability dye Ghost Dye^™^ Violet 780 (Tonbo Bioscience, San Diego, CA, USA, cat. 13-0865-T100). All samples were analyzed on a BD FACSAria II flow cytometer. Data was analyzed using FlowJo software (v.10.10.0).

### Mouse HSPC isolation and culture.

Bone marrow was harvested from B6.SJL-Ptprc^a^ Pepc^b^/BoyJ mice and HSPCs were isolated using the CD117 MicroBeads, mouse kit (Miltenyi Biotec, cat: 130-091-224). Cells were cultured in CellBIND plates (Corning, Corning, NY, USA, cat: 3337) in Nutrient Mixture F-12 Ham (Sigma-Aldrich, cat: N6658) supplemented with 1X penicillin-streptomycin-glutamine (Gibco, Waltham, MA, USA, cat: 10378-016), 1X Insulin-transferrin-selenium-ethanolamine (ITS-X) (Gibco, cat: 51500-056), 1 mg/mL polyvinyl alcohol (PVA) (Sigma-Aldrich, cat: P8136), 100 ng/mL TPO (Peprotech, cat: 315-14 or Biolegend cat: 718102), and 10 ng/mL SCF (Peprotech, cat: 250-03), based on a previously published protocol^41^, for 2-4 weeks. Prior to editing, cultures were checked for purity via flow cytometry. Cells were blocked for non-specific binding using FcR Blocking Reagent for human (Miltenyi Biotec, cat.: 130-059-901) 10 min at room temperature and then stained for 30 min at 4°C with the following antibody cocktail: anti-mouse Lineage cocktail BV421 (Biolegend, cat.: 133311), anti-mouse Sca-1 BV650 (Biolegend, cat.: 108143), anti-mouse CD117 (c-Kit) BV786 (Biolegend, cat.: 105841), anti-mouse CD150 PE (Biolegen, cat.: 115904), and LIVE/Dead staining solution (Invitrogen, cat.: L23105). All samples were analyzed on a BD FACSAria II flow cytometer. Data was analyzed using FlowJo software (v.10.10.0).

### Genome editing of primary cells.

Synthetic chemically modified sgRNAs were purchased from Synthego (Redwood City, CA, USA) or TriLink Biotechnologies (San Diego, CA, USA). Chemical modifications were comprised of 2′-O-methyl-3′-phosphorothioate at the three terminal nucleotides of the 5′ end and the second, third, and fourth bases from the 3′ end as described previously^72^. The target sequence for the sgRNAs are as follows: *CCR5*-sgRNA^22,23^ 5′-TGACATCAATTATTATACAT-3′ and *Rosa26*-sgRNA^42-44^ 5′- ACTCCAGTCTTTCTAGAAGA-3′.

HiFi Cas9 protein was purchased from Aldevron (Fargo, ND, USA cat:9214). RNPs were complexed at a Cas9:sgRNA molar ratio of 1:2.5 at room temperature for 15-30 min. Cells were resuspended in P3 buffer (Lonza, Basel, Switzerland, cat.: V4XP-3032) with complexed RNPs and electroporated using the Lonza 4D Nucleofector and 4D-Nucleofector X Unit (program DZ-100 for human HSPCs, EO-117 for B cells, and CA-137 for mouse HSPCs). For some editing experiments, 5 μM of HDR Enhancer Protein (IDT, San Jose, CA) was added to the electroporation mixture prior to electroporation. Immediately following electroporation, AAV6 was dispensed onto cells based on titers determined by ddPCR (0.625-2.5 × 10^3^ vg/cell for human and mouse HSPCs, 1-2.5 × 10^4^ vg/cell for B cells). For mouse HSPCs and B cells, cells were plated in ~100 μL of serum-free media with AAV6 in a 96 well plate for 3–4 hours prior to replating. For some editing experiments, cells were incubated with 0.5 μM (human HSPCs) or 1 μM (mouse HSPCs) of AZD7648 (Selleck Chemicals, Houston, TX, cat.: S8843) for 24 h, as previously described^29^.

### Colony forming unit (CFU) assay.

Two days post-electroporation 500 HSPCs were plated in each well of a SmartDish 6-well plate (STEMCELL Technologies, Vancouver, Canada, cat.: 27370) containing MethoCult H4434 Classic (STEMCELL Technologies, cat.: 04444). After 14 days, the wells were imaged using the STEMvision Hematopoietic Colony Counter (STEMCELL Technologies). Colonies were counted and scored with manual correction to determine the number of BFU-E, CFU-GM, and CFU-GEMM colonies.

#### Targeted integration analysis by ddPCR.

Genomic DNA was extracted from cells using QuickExtract DNA extraction solution (Biosearch Technologies, Hoddesdon, UK, cat.: QE09050). To quantify knock-in alleles via ddPCR, we employed *HBA1* specific in-out PCR primers and a probe corresponding to the expected knock-in event (1:3.6 primer to probe ratio). We also used an established genomic DNA reference at the *CCRL2* locus.^35^ The ddPCR reaction was prepared and underwent droplet generation following the manufacturer’s instructions with a Bio-Rad QX200 ddPCR machine (Bio-Rad, Hercules, CA).

Thermocycler settings were as follows: 95°C (10 min, 1°C/s ramp), 94°C (30 s, 1°C/s ramp), 57.7°C (30 s, 1°C/s ramp), 72°C (2 min, 1°C/s ramp), return to step 2 for 50 cycles, and 98°C (10 min, 1°C/s ramp). Analysis of droplet samples was then performed using the QX200 Droplet Digital PCR System (Bio-Rad). We divided the copies/μL to determine the frequency of HDR (%): HDR (FAM) / REF (HEX). The following primers and probes were used in the ddPCR reaction:

CCR5:

Forward Primer (FP): 5’-GGGAGGATTGGGAAGACA-3’

Reverse Primer (RP): 5’-AGGTGTTCAGGAGAAGGACA-3’

Probe: 5’-6-FAM/AGCAGGCATGCTGGGGATGCGGTGG/3IABkFQ-3’

CCRL2 (reference):

FP: 5’-GCTGTATGAATCCAGGTCC-3’,

RP: 5’- CCTCCTGGCTGAGAAAAAG 3’

Probe: 5’-HEX/TGTTTCCTC/ZEN/CAGGATAAGGCAGCTGT/3IABkFQ-3’

*Rosa26* (targeted integration)^44^*:*

FP: 5’-AAGGGGGAGGATTGGGAAGA-3’,

RP: 5’-ACAGCCTCGATTTGTGGTGT-3’

Probe: 5’-6-FAM/CATGCTGGGGATGCGGTGGG/3IABkFQ-3’

*Rosa26* (reference):

FP: 5’-AAGCCACTGACTATGGTGCC-3’,

RP: 5’-CTCAGAAGCAGAAGCATCCCT-3’

Probe: 5’-6-FAM/TGTTCTCAAAGGAAGGATTGTCTGTGC/3IABkFQ-3’

### Enzyme-linked immunosorbent assay (ELISA) to detect antibodies.

For detection of therapeutic antibodies, antigen specific ELISA was used. For detection of total IgG in B cell supernatant, anti-IgG Fc ELISA was used. NUNC Maxi Sorp plates (Thermo Fisher Scientific cat: 50-314-51) were coated with 4 μg/mL streptavidin (Thermo Fisher Scientific, cat: 21122) for 1 h at room temperature. Plates were then washed 3 times with MilliQ water and then blocked with ChonBlock (Thermo Fisher Scientificc, cat.: 50-152-6971) overnight at 4°C. The next day, plates were coated with 1 μg/mL of antigen (biotinylated human PCSK9 protein or biotinylated human TNF-alpha protein, Acro Biosystems, cat: PC9-H82E7, TNA-H82E3) or biotinylated IgG Fc protein (Fortis Life Sciences, Waltham, MA, USA, cat: A80-104B) for 1 h at room temperature. Plates were then washed with 0.05% PBS-Tween (PBS-T). Media or serum samples were diluted in PBS containing 0.1% BSA and 0.05% Tween-20 and then incubated on the plate for 1 h at room temperature. Plates were washed with PBS-T, followed by incubation with the secondary antibody, horseradish peroxidase (HRP)–conjugated goat anti-human IgGFc antibody (Fortis Life Sciences, cat.: A80-104A) at a 1:2500 dilution. Plates were washed with PBS-T and detection was performed with the 1-Step^™^ TMB Substrate Kit (Thermo Fisher Scientific cat.: 34021) and quenched with 3M H_2_SO_4_. Plates were read at 450 nm using a Molecular Devices SpectraMax M3 plate reader with SoftMax Pro software. Standard curves were created using purified linker versions of each antibody or purified human IgG from normal serum (Bethyl, Montgomery, TX, USA, cat.: P80111). Results were analyzed using GraphPad Prism v10 software to calculate a standard curve using a 4-parameter sigmoidal algorithm.

### Western blot for purified antibodies.

Purified antibodies (0.5 μg each) were denatured under reducing conditions by adding 4X Laemmli sample buffer (Bio-Rad, cat.: 1610747) and 2-mercaptoethanol and heated at 100°C for 5 min. Samples were loaded onto a 4-15% MiniPROTEAN TGX precast protein gel (Bio-Rad, cat.: 4561084). Following electrophoresis, protein from gel was transferred to a PVDF membrane using the Trans-Blot Turbo Transfer System (Bio-Rad, cat.: 1704150) and the membrane was blocked using Blotting-Grade Blocker (Bio-Rad, cat.: 1706404) in phosphate-buffered saline with 0.02% Tween 20 (PBS-T) for 30 min at room temperature. The membrane was then incubated in 1:5000 primary antibody (HRP-conjugated Goat pAb to human IgG, Abcam, cat.: 7153) overnight at 4°C. The next day, the membrane was washed three times with PBS-T. SuperSignal^™^ West Pico PLUS Chemiluminescent Substrate (Thermo Fisher Scientific, cat.: 34579) was used for detection and the membrane was imaged using a Bio-Rad ChemiDoc XRS+ gel imager using ImageLab software.

### Low-density lipoprotein receptor (LDLR) expression via flow cytometry.

2.5×10^5^ HepG2 (ATCC, Manassas, VA, USA, cat.: HB-8065) cells were seeded in 6 well plates. The next day, purified anti-PCSK9 antibodies were applied to cells at 10 ug/mL in the media and allowed to incubate for 48 hours. Media was then removed and cells were washed once with PBS and detached using Accutase cell detachment reagent (Innovative Cell Technologies, San Diego, CA, USA, cat.: AT104). Cells were then collected and blocked for non-specific binding using FcR Blocking Reagent for human (Miltenyi Biotec, cat.: 130-059-901) for 10 min at room temperature. Cells were then stained with anti-human LDLR BV421 (BD Biosciences, Franklin Lakes, NJ, USA, cat.: 744847) and viability dye Ghost Dye^™^ Violet 780 (Tonbo Bioscience, San Diego, CA, USA, cat. 13-0865-T100) for 20 minutes at 4°C. Samples were analyzed on a LSR Fortessa flow cytometer (BD Biosciences). Data was analyzed using FlowJo software (v.10.10.0).

### NF-κB reporter assay for TNFα acitivity.

Purified anti-TNFα antibodies or B cell supernatant was incubated with recombinant human TNFα (R&D Systems, Minneapolis, MN, USA, cat.: 210-TA) for 30 minutes at room temperature in a 96-well plate. Following incubation, 1×10^4^ THP-1 NF-κB-Luc2 reporter cells (ATCC, cat.: TIB-202-NFkB-LUC2) were added to each well and incubated for 2 hours at 37°C. Cells were lysed using Britelite plus reagent (Revvity Health Sciences, Waltham, MA, USA, cat.: 6066766) and relative luminescence units (RLU) was determined using a Synergy H1 plate reader (BioTek, Winooski, VT, USA).

### Xenotransplantation of human HSPCs in immunodeficient mice.

All mice were housed at Stanford University with a 12/12h light/dark cycle at an ambient temperature of 22°C and 50% humidity. All experiments were completed under the Administrative Panel on Laboratory Animal Care (APLAC Protocol #25065).Six- to eight-week-old NOD.Cg-Prkdc^scid^Il2rg^tm1Wjl^/SzJ (NSG) mice (Jackson Laboratories, Strain: 005557) were irradiated with 2 Gy and then transplanted with 5 × 10^5^ mock (electroporation only) or genome-edited cord-blood derived CD34^+^ HSPCs via retro-orbital injection. Human engraftment in bone marrow and spleen was assessed at 16-weeks post-transplantation. Bone marrow mononuclear cells were isolated via Ficoll gradient centrifugation. Spleen samples were treated with RBC Lysis Buffer (IBI Scientific, Dubuque, IA, USA, cat.: IB47620) to eliminate mature red blood cells. Samples were blocked for non-specific binding using FcR Blocking Reagent for human (Miltenyi Biotec, cat.: 130-059-901) and mouse (Miltenyi Biotec, cat.: 130-092-575) for 10 min at room temperature. Samples were then stained for 30 min at 4°C with the following antibody cocktail: anti-mouse CD45.1 BV650 (Biolegend, cat.: 110735), anti-mouse TER-119 PE-Cy5 (eBiosciences, San Diego, CA, USA, cat.: 15-5921-82), anti-human CD45 PacBlue (Biolegend, cat.: 368540), anti-human HLA-ABC APC-Cy7 (Biolegend, cat. 311426), anti-human CD33 PE (BD, cat.: 555450), anti-human CD19 APC (Biolegend, cat.: 302212), anti-human CD3 FITC (Biolegend, cat.: 300305), and viability dye Ghost Dye_™_ Violet 540 (Tonbo Bioscience, San Diego, CA, USA, cat. 13-0879-T100). Samples were analyzed on a CytoFLEX flow cytometer (Beckman-Coulter, Indianapolis, IN, USA). Data was analyzed using FlowJo software (v.10.10.0).

### Transplantation of murine HSPCs in immunocompetent mice.

Eight to twelve-week old C57BL/6 mice (Jackson Laboratories, Strain: 000664) were irradiated with 9.5 Gy and then transplanted with 1×10^6^ mock (electroporation only) or genome-edited murine HSPCs along with 2.5×10^5^ whole bone marrow support cells previously harvested from euthanized 8- to 12-week-old CD45.1/CD45.2 mice via retroorbital injection. Peripheral blood samples were collected via retro-orbital blood collection 4-, 8-, and 12- weeks post-transplantation. At 16-weeks post-transplantation, mice were euthanized, and bone marrow, spleen, and peripheral blood were harvested. All samples were treated with RBC Lysis Buffer (IBI Scientific, cat.: IB47620) to eliminate mature red blood cells. Samples were blocked for non-specific binding using FcR Blocking Reagent for mouse (Miltenyi Biotec, cat.: 130-092-575) for 10 min at room temperature and then stained for 30 min at 4°C with the following antibody cocktail to evaluate engraftment: anti-mouse CD45.2 BV421 (Biolegend, cat.: 109831), anti-mouse CD45.1 BV711 (Biolegend, cat.: 110739), anti-mouse CD11b PE (eBiosciences, cat.: 12-0112-82), anti-mouse Ly-6G/Ly-6C PE (eBiosciences, cat.: 12-5931-82), anti-mouse B220 APC-Cy7 (BD Biosciences, cat.: 561102), anti-mouse CD4 APC (eBiosciences, cat.: 17-0042-82), anti-mouse CD8 APC (eBiosciences, cat.: 17-0081-82), and LIVE/Dead staining solution (Invitrogen, cat.: L23105). All samples were analyzed on a BD FACSAria II flow cytometer and B220^+^ cells were sorted from spleen samples (BD Biosciences, Franklin Lakes, NJ, USA). Data was analyzed using FlowJo software (v.10.10.0).

### Immunophenotyping of murine B cells.

Bone marrow and spleen were isolated from C57BL/6 mice and prepared as described previously. Samples were then blocked for non-specific binding using FcR Blocking Reagent for mouse (Miltenyi Biotec, cat.: 130-092-575) for 10 min at room temperature and then stained for 30 min at 4°C with the following antibody cocktail: anti-mouse CD45.2 PE-Cy7 (Biolegend, cat.: 109830), anti-mouse CD45.1 BV650 (Biolegend, cat.: 110736), anti-mouse B220 APC-Cy7 (BD Biosciences, cat.: 561102), anti-mouse CD43 APC (BD Biosciences, cat.: 560663), anti-mouse IgD BV711 (BD Biosciences, cat.:564275), anti-mouse IgM FITC (BD Biosciences, cat.: 553437), anti-mouse CD24 BV421 (BD Biosciences, cat.: 562563), anti-mouse CD249 PE (BD Biosciences, cat.: 553735), and LIVE/Dead staining solution (Invitrogen, cat.: L23105). Samples were analyzed on a BD FACSAria II flow cytometer.

### B-cell receptor (BCR) repertoire analysis.

B220^+^ cells were sorted from spleen samples of transplanted mice at 16 weeks post-transplant and RNA was extracted using the RNeasy Plus Micro Kit (Qiagen, Netherlands, cat.: 74034). SMART-Seq Mouse BCR kit (Takara, cat.: 634352) was used to sequence CDR3 regions and the Cogent NGS Immune Profiler software (v2.0, Takara) was used to reconstruct BCR sequences and assign V(D)J gene segments. Only productive BCR rearrangements were retained for analysis. PCR duplicates were collapsed using unique molecular identifiers (UMIs), and reconstructed sequences supported by at least one UMI were retained. Filtered TRUST4 outputs were exported in AIRR format and used for downstream repertoire analysis with Immunarch^73^. Clonotypes were defined by identical CDR3 amino acid sequences with shared V and J gene usage. Distributions of CDR3 nucleotide and amino acid lengths were evaluated across unique clonotypes in three mouse groups. V and J gene usage frequencies were calculated based on relative segment usage within each group. Repertoire diversity was quantified using the D50 metric, defined as the minimum number of unique clonotypes accounting for at least 50% of total sequencing reads. Statistical comparisons across groups were performed using the Kruskal–Wallis test, with P values computed within the Immunarch pipeline and adjusted for multiple testing using the Holm–Bonferroni correction.

### Statistical analysis.

GraphPad Prism v10 software was used for all statistical analysis unless otherwise noted.
